# Improving Early Identification and Access to Diagnosis of Autism Spectrum Disorder in Toddlers in a Culturally Diverse Community with the Rapid Interactive screening Test for Autism in Toddlers

**DOI:** 10.1007/s10803-020-04851-3

**Published:** 2021-01-09

**Authors:** Roula Choueiri, Asher Lindenbaum, Manasa Ravi, William Robsky, Julie Flahive, William Garrison

**Affiliations:** 1 Developmental and Behavioral Pediatrics, University of Massachusetts Medical School/Children’s Medical Center, 55 Lake Avenue North, Worcester, MA 01655 USA; 2 Peachtree Pediatric Psychology, Atlanta, GA USA; 3 Department of Population and Quantitative Health Sciences, University of Massachusetts Medical School, Worcester, MA USA

**Keywords:** Screening, Autism, Interactive, Toddlers, Access, Underserved, Cultural diversity, Early intervention, Community, RITA-T (Rapid Interactive Screening Test of Autism in Toddlers)

## Abstract

The objective of this study was to test a screening model that employs the Rapid Interactive Screening Test for Autism in Toddlers (RITA-T), in an underserved community to improve ASD detection. We collaborated with a large Early Intervention (EI) program and trained 4 providers reliably on the RITA-T. Toddlers received the Modified Checklist for Autism in Toddlers (MCHAT-R/F), the RITA-T, developmental and autism testing, and a best-estimate clinical diagnosis. Eighty-One toddlers were enrolled: 57 with ASD and 24 with Developmental Delay (DD) non-ASD. Wait-time for diagnosis was on average 6 weeks. The RITA-T correlated highly with autism measures and EI staff integrated this model easily. The RITA-T significantly improved the identification and wait time for ASD in this underserved community.

## Introduction

The Early Identification of Autism Spectrum Disorder (ASD) is an evolving research area. Benefits of early intervention on improving the course and outcomes of the disorder are well known (Suma et al. 2016). However, ASD is still not detected early enough in the United States (Baio et al. 2018), and especially for children from culturally diverse and underserved communities (Elder et al. 2016; Zuckerman et al. 2014). While there are significant basic research advances occurring, such as eye-tracking studies (Wan et al. 2018), electroencephalogram (EEG) markers (Bosl et al. 2011), and genetic or biochemical biomarkers (Bridgemohan et al. 2019; Yuen et al. 2017), these approaches are still far from clinical application and generalization. Improving the early identification of ASD remains largely a clinical challenge.

The American Academy of Pediatrics continues to support screening for ASD in primary care (Johnson and Myers 2007). Other programs, such as the Centers for Disease Control (CDC) “ Learn the Signs. Act Early” Campaign, launched in 2004, have developed educational material targeting early childhood providers and parents. These materials are designed to improve developmental monitoring and the detection of early signs of ASD (Gadomski et al. 2018; “Learn the Signs. Act Early” CDC website 2019). Improving the early identification of ASD and access to diagnosticians, are urgent public health goals, as they will lead to early intensive interventions to improve outcomes. Currently, ASD diagnosticians are in very limited supply and overwhelmed with the volume of referrals. Patient wait times for evaluations vary by state, with reports of 12 months or more in many areas of the United States (Austin et al. 2016). Referrals and wait times are typically further increased among culturally diverse children with limited options through health insurance and other obstacles to their care (Rea et al. 2019). In addition, wait lists, for diagnostic evaluations in toddlers with ASD, include other young children who present with developmental delay without the presence of ASD. The merging of children, who do not have ASD with those who do, dramatically slows the process for those who will need more specialized intervention than existing early childhood programs can provide.

In our clinical center, the average wait time for a toddler aged 18–36 months referred for evaluation because of concerns of ASD, varies between 3 and 5 months with an average wait of 4 months or 16 weeks. To improve access and early identification of ASD in our culturally diverse community, we implemented and assessed a clinical approach that utilizes a two-level ASD screening model, in combination with a close partnership with the largest Early Intervention (EI) program in our region.

### Two-Level Screening Model

A two-level ASD screening model concept is not new and was first described by Oosterling et al. (2010). It integrates a Level 1or universal screen, and a Level 2 or more disorder-specific screening test. A Level 1 screen, such as the Modified Checklist for Autism in Toddlers-Revised with Follow-up Interview (MCHAT-R/F; Robins et al. 2013), will identify those at an increased risk for a developmental delay or disorder from the general population. The Positive Predictive Value (PPV) of the MCHAT-R/F is close to 98% for developmental delay and 54% for ASD in a low-risk group such as during well child visits. However, there are no consistent patterns in the administration of the MCHAT R/F in primary care. It is also likely that the MCHAT-R/F alone will miss a substantial number of children with ASD when questions are not reviewed with parents. A recent study looking at ASD screening by family practitioners (Carbone et al. 2020) showed that: Hispanic children were less likely to be screened, family practitioners were less likely to complete a screen, screenings were not always scored as per the recommendations, and even when there was a positive screen, referrals to an ASD evaluation did not happen immediately. In the Carbone et al. (2020) study, the PPV of the MCHAT-R/F for an ASD diagnosis was 17.8%, much lower than the 54% in the initial study of the MCHAT-R/F (Robins et al. 2013). Questionnaires are also difficult to complete for first time parents, parents whose child has inconsistent skills, or parents from a different culture as they may answer incorrectly (Choueiri and Wagner 2015; Stone et al. 2004).

Toddlers in Early Intervention (EI) have already been identified at risk for delays. Thus, administering the MCHAT-R/F to this group in EI will have higher PPV for an ASD with reported PPV of 61–79% (Pandey et al. 2008). After identification of a group as at risk with a Level 1 measure, administering then an interactive Level 2 measure specifically designed to evaluate ASD signs and symptoms, will identify those at real elevated risk for ASD. Figure one summarizes this model.

**Fig. 1:**
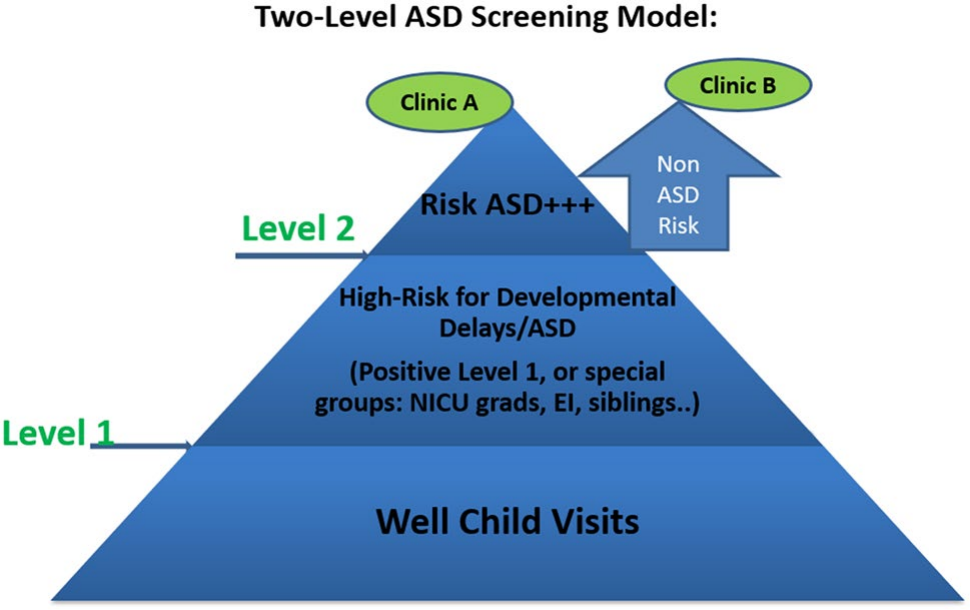
Two-Level ASD screening model

In this study, we integrated the Rapid Interactive Screening Test for Autism in Toddlers (RITA-T) as a Level -2 screening tool (Choueiri and Wagner 2015). This two-level screening model improves the triage process for those at risk for ASD, by referring them sooner to more appropriately focused ASD evaluation clinics (Fig. 1). This model could also act to reduce overall wait time for both types of children (non ASD risk and elevated ASD risk). In addition, partnering with EI providers within a community or region is key in effective screening of an already existing child population at elevated risk for ASD. This partnership has also added benefits in that if a primary care physician does refer to EI because of developmental concerns, at least the child can be followed more consistently and screened then more efficiently. In Carbone et al. (2020) recent review of primary care practices, half of the primary care practices surveyed referred to an EI program although their screening for autism was not consistent.

An interactive screening tool is preferred to a parent or provider questionnaire alone at this age as it operates to more directly trigger core social communication behavioral signs that are delayed. It also allows the provider to directly observe and rate the essential features of ASD in young toddlers (Choueiri and Wagner 2015; Lemay et al. 2018, 2020). It is important that a Level 2 screening method is easy to learn, demonstrably reliable and valid. Such a screening tool should be concise, inexpensive and integrate effectively into clinical workflows in a range of real-life settings where children can be more readily screened. There is high value for a Level 2 screening test that is non-language dependent, and that can show similar results across different cultures (Rea et al. 2019). The RITA-T offers each of these features.

### The Rapid Interactive Screening Test for Autism in Toddlers (RITA-T)

We have previously reported a pilot study on the Rapid Interactive Screening Test for Autism in Toddlers (Choueiri and Wagner 2015). The RITA-T includes nine interactive activities that evaluate key social communication skills in toddlers with elevated risk for ASD, such as *joint attention*, *social awareness*, and *human agency*. The associated scoring sheet, manual and training have been validated. An online training course, as well as in-person workshops, have been developed to facilitate training and improve rater reliability after initial training (RITA-T 2015). Our initial study showed that the RITA-T scores correlated highly with best clinical-estimate diagnoses of autism and non-autism/developmental delay and no developmental concerns (Choueiri and Wagner 2015). It also correlated highly with autism diagnostic measures such as the Autism Diagnostic Observation Schedule (Lord and Rutter 2012) and the Diagnostic and Statistical Manual, Fifth Edition (DSM-5) criteria for ASD (American Psychiatric Association 2013). These findings were replicated by Lemay et al.’s study of the RITA-T in 239 toddlers (Lemay et al. 2020). The RITA-T training for providers is, on average, 3 h long to establish reliability, and its administration and scoring for participants is approximately 10 min in duration, making it simple to train and integrate into various settings. The administration of the RITA-T does not rely on language, which makes it more easily applicable to diverse cultural settings.

Over the last 3 years, the RITA-T has been integrated within pilot models that are part of ongoing projects in different clinical and community settings and cultures. It has been empirically evaluated at Alberta Children’s Hospital in Calgary, Canada (Lemay et al. 2018, 2020), where Lemay and his team integrated it in their effort to improve their triaging of referrals to their tertiary care center. They showed a much-improved wait time and excellent psychometrics in the identification of ASD, while improving costs and reduction of clinician work time through better triage, which in turn allowed more rapid and expanded access. Recently, it has been validated in a small pilot study in a Lebanese population (Yassin et al. 2020) and its translation into other different languages is ongoing with plans to implement the RITA-T in other countries.

In this current study our objective was to test a screening model that employs the RITA-T in a diverse and underserved community to improve ASD detection. We also looked at the RITA-T psychometrics in this setting.

## Methods

The research team reached out to the THOM Early Intervention (EI) Program, the largest EI program in Worcester, MA where this study was completed. The town of Worcester, MA has a culturally diverse population, with 21% of the population living below the federal poverty level and close to 35% with a primary language other than English (US Census Bureau QuickFacts 2019). We trained four THOM EI providers reliably on the RITA-T. The training was completed in person and inter-rater reliability was calculated. THOM was starting an internal training for their staff on the MCHAT-R/F at the same time. Toddlers, 18–36 months of age, enrolling in EI or already in EI, were administered the MCHAT-R/F as part of their evaluation or re-evaluation within 6 months, or if their provider had concerns for ASD. The MCHAT-R/F was administered in an interactive way, with parent and provider discussing each question. For this study, after consenting the families as per our Institutional Review Board approved protocol, when the MCHAT-R/F had a score above 2, or if the child’s providers had concerns for ASD, the RITA-T was administered by one of the trained providers. Results of the MCHAT-R/F and the RITA-T were sent to the research assistant (RA) in the research team in a sealed envelope. Those results were then entered in the study database without the PI or the research psychologist being aware of them. The family’s EI provider then began the conversation with the family about concerns for ASD. Children and their families were then referred to the research psychologist on the team, who was blinded to the results of the MCHAT-R/F and the RITA-T. The psychologist obtained a brief developmental history, observed the DSM-5 criteria for ASD and administered the Autism Diagnostic Observation Schedule, Second Edition (ADOS-2) (Lord and Rutter 2012) as well as the Mullen Scales of Early Learning (MSEL) (Mullen 1995). The ADOS-2 Toddler module or Module 1 was administered depending on the age of the child. The EI provider and the RA scheduled the child and his/her family for an appointment with the PI after the child was evaluated by the research psychologist.

The research psychologist met with the Principal Investigator (PI) on a weekly basis and reviewed autism and developmental testing with the PI without disclosure of previous MCHAT R/F and RITA-T results. A “best estimate diagnosis” was made based on diagnostic tests and history obtained by the research psychologist. Two groups were identified: toddlers with ASD diagnosis and those with a non-ASD/Developmental Delay (non-ASD/DD) diagnosis. Autism and developmental testing results and algorithms scoring documents were shared with the RA who then entered them in the database. At the time of the appointment with the PI, the PI met with the family, child, EI provider, and an interpreter as needed. The PI then completed a medical and developmental history, observed behavior and play, completed a physical exam and reviewed previous test results and current observations with the family. All previous testing was then available to the PI, including previous results of the MCHAT R/F and the RITA-T. The PI then reviewed and discussed diagnoses, recommendations and services while providing a letter to initiate services. The best estimate diagnosis was provided to the RA who entered it in the database. A follow up was arranged within 1–3 months with a family support specialist in our center.

We collected data on time-in-days between referral from EI after administration of RITA-T to meeting with the PI for review of testing and final diagnosis and compared this with wait time to be evaluated if a toddler was referred outside of this model to our center. To further enrich the sample of toddlers enrolled from EI, we also enrolled 12 toddlers, ages 18–36 months, from EI who had no concerns for ASD. They were administered the RITA-T, the Battelle Developmental Inventory, Second Edition (BDI-2) (Newborg 2005) as per EI protocol, and a brief developmental history was obtained. In addition, a DSM-5 checklist and a MCHAT-R/F were completed by their EI provider and family respectively.

Pediatricians in two large practices in Worcester, one hospital based and one large private practice, were made aware about this project. Both practices referred children with developmental concerns to THOM EI. Both practices administered the MCHAT-R/F at 18 and 24 months and usually referred as well to our center for evaluations. When toddlers were administered the RITA-T by EI trained providers, their pediatrician was made aware of the results and the referral for an evaluation by the research team.

### Statistical Methods

Demographics and test scores were compared between ASD and Non-ASD/DD groups. Percentages were reported for categorical variables, for normally distributed continuous variables, means and standard deviations (SD) were calculated, and medians and interquartile ranges (IQR) were computed for non-normally distributed variables. A Chi-Square test was used to compare percentages between the two groups. For continuous variables, a t-test was performed to compare means of normally distributed variables, and the Wilcoxon rank-sum test was used for comparing non-normally distributed variables between the two groups. An unadjusted logistic regression model was used to assess the relationship between RITA-T score and ASD diagnosis. An alpha level of 0.05 was used to determine statistical significance. Sensitivity, specificity, positive predictive value (PPV), and negative predictive value (NPV) were calculated at different cut points of the RITA-T total score. A scatterplot of RITA-T total scores was plotted by random ID assignment of each patient. The scores were sorted from highest to lowest and plotted by ID and divided into ASD versus Non ASD. Analyses were conducted using SAS 9.4 software (SAS Institute Inc, Cary, NC).

## Results

Over a period of 12 months, 81 toddlers were enrolled in this project. Of those, 57 received a diagnosis of ASD and 24 had a diagnosis of non-ASD/DD. The groups had similar gender distribution and age in months; race and income were representative of the area where this study was completed and were similar in the two groups. The RITA-T mean scores were significantly higher in the ASD group than in the non-ASD/DD group with a mean score of 20.1 (SD = 3.9) in those diagnosed with ASD and a mean score of 9.7 (SD = 2.9) in those diagnosed as non-ASD/DD (p < 0.0001). The MSEL composite scores were also significantly different between groups, which can be interpreted as meaning that those ultimately diagnosed with ASD demonstrated greater delays than those without a diagnosis of ASD. The ADOS-2 total scores were significantly different between both groups [ASD: 17.4 (SD = 3.9) vs. non-ASD/DD/: 3.9 (SD = 2.8), p < 0.0001]. The MCHAT R/F mean scores were significantly different between those in the ASD group vs. those in the non-ASD/DD group. Interestingly, among those with MCHAT R/F > 2, 50 out from the 57 children in this study were diagnosed with ASD yielding a PPV of 87.7% which is much higher than the previously reported PPV in a high-risk group of 61–79% (Pandey et al.^2008^). Furthermore, the MCHAT R/F scores seemed to be associated with a diagnosis of ASD in this current study independently from the RITA-T scores (Table 1).

**Table 1:** Demographics and test results on all study patients

	ASD	Non ASD	p-value
Demographics	(n = 57)	(n = 24)	
Female sex, n (%)	9 (16)	7 (29)	0.22
Age, mo. mean (SD)	27.3 (5.1)	27.8 (4.3)	0.68
Race, n (%)			0.32
White	29 (51)	18 (75)	
Black/African American	10 (18)	2 (8.3)	
Hispanic	13 (23)	3 (13)	
Asian	5 (8.8)	1 (4.2)	
Income in $1000′s, median (IQR)	72 (59, 87)	81 (63, 10)	0.22
Test scores, mean (SD)			
RITA-T, total score	20.1 (3.9)	9.7 (2.9)	< 0.0001
M-CHAT, total score	8.6 (3.8)	0.1 (0.3)	< 0.0001
MSEL RL, T score	24.0 (8.5)	46.4 (12.8)	< 0.0001
MSEL EL, T score	24.3 (5.8)	34.4 (12.4)	< 0.0001
MSEL VR, T score	28.2 (8.7)	48.2 (15.4)	< 0.0001
MSEL FM, T score	31.1 (10.6)	48.3 (15.2)	0.002
Mullen cognitive T score sum	105.6 (30.3)	177.3 (44.5)	< 0.0001
Mullen calculated cognitive sum	107.6 (25.5)	96 (95.8)	0.40
Mullen Early learning comp standard	59.2 (10.5)	89.6 (21.1)	< 0.0001
Battelle Ad	–	90.4 (10.1)	
Battelle Pe_So	–	92.4 (10.9)	
Battelle Comm	–	77.9 (15.1)	
Battelle Mot	–	102 (14.8)	
Battelle Cog	–	91.1 (11)	
ADOS2-toddler module*	17.4 (3.9)	3.9 (2.8)	< 0.0001
ADOS2-module 1**	14.2 (4.5)	3.8 (1.7)	< 0.0001
DSM-5, total hits***	4.3 (1.1)	0.67 (0.92)	< 0.0001

*ASD n = 39, non-ASD n = 8

**ASD n = 18, non-ASD n = 4

***ASD n = 57, non-ASD n = 24

*ASD* autism spectrum disorder, *DD* developmental delays, *IQR* interquartile range

In looking at the relationship between the ADOS-2 and DSM-5 with the RITA-T, we assessed the correlation between their scores. The Pearson correlation coefficient for ADOS (either Module 1 or Toddler) by RITA-T was 0.70 (p < 0.0001). This illustrates that as the RITA-T total score was increased, the ADOS-2 total score also increased, and the relationship between the two scores was moderately strong. In order to investigate the association between ASD diagnosis and RITA-T score, we modeled diagnosis as a function of RITA-T score. Using unadjusted logistic regression to predict ASD diagnosis, we found that the odds of being diagnosed with ASD is almost 3 times as likely when compared to non-ASD/DD [OR 2.96, 95% CI (1.42, 6.15)].

To determine the best cut-point for RITA-T in determining ASD diagnosis, we investigated sensitivity, specificity, positive predictive value (PPV) and negative predictive value (NPV). These statistics were calculated for each possible RITA-T score (Table 2).

**Table 2:** RITA-T sensitivity, specificity, PPV, and NPV (n = 81)

RITA-T total score	Sensitivity	Specificity	PPV	NPV
3	1	0.04	0.71	1
4	1	0.04	0.71	1
5	1	0.04	0.71	1
6	1	0.13	0.73	1
7	1	0.17	0.74	1
8	1	0.42	0.80	1
9	1	0.54	0.84	1
10	1	0.54	0.84	1
11	1	0.67	0.88	1
12	0.98	0.83	0.93	0.95
13	0.93	0.96	0.98	0.85
14	0.88	0.96	0.98	0.77
15	0.82	0.96	0.98	0.70
16	0.82	1	1	0.71
17	0.77	1	1	0.65
18	0.68	1	1	0.57
19	0.60	1	1	0.51
20	0.51	1	1	0.46
21	0.39	1	1	0.41
22	0.26	1	1	0.36
23	0.19	1	1	0.34
24	0.14	1	1	0.33
25	0.07	1	1	0.31
26	0.05	1	1	0.31
27	0.02	1	1	0.30
28	0	1	–	0.30

In our sample, a cut-off score of 16 had best PPV (1) and specificity (1), meaning that all children with a score higher than 16 were ultimately diagnosed with ASD.

Wait time between referral to final diagnosis varied between 14 to 105 days with a mean of 6 weeks. Wait time in this center for those 18–36 months referred with a question of ASD and not part of this current model varied between 3 and 5 months with an average of 4 months or 16 weeks. We also looked at inter-rater reliability and asked the 4 EI providers to each independently score 3 videos of administration of the RITA-T. Scores were compared with the PI’s scores and results showed an inter-rater reliability of 80% for total scores among each provider and among each provider and the PI.

A short survey was completed with EI providers about the usefulness of this model and for feedback. The RITA-T allowed providers to: (1) evaluate for the constructs delayed in ASD; and (2) empowered them to start a conversation with the family regarding concerns of ASD.

## Discussion

This is the third in a series of studies to determine the RITA-T sensitivity, specificity and predictive value. The initial study (Choueiri and Wagner 2015) was exploratory and hospital based entirely. That study included 61 toddlers, 19 of which had no developmental concerns at the time of the study. The second RITA-T study looked at the psychometrics of the RITA-T in 239 toddlers within a hospital based triaging model including the RITA-T (Lemay et al. 2018, 2020). In the current study, we included the RITA-T in a two-level screening model and partnered with an Early Intervention program in this culturally diverse community. Specifically, our goals were to study the feasibility and improved access in those children 18–36 months old with concerns for ASD. The current study showed improvement with access to diagnosis with an average wait time of 6 weeks compared to an average of 16 weeks in those children referred for an ASD evaluation who were not part of this model. The RITA-T identified those at-risk for ASD and diverted them in a timely manner for a formal ASD diagnostic evaluation and subsequent specialized services. Reliable training and inter-rater reliability were achieved after 3 h of in-person training. In addition, the RITA-T correlated strongly with autism diagnostic measures, including the ADOS-2, DSM-5 criteria for ASD, and clinician judgment.

It is interesting to note the difference in developmental levels between both groups. The MSEL was used as a measure of developmental levels. This difference could be because the MSEL relies on language and the child to be focused during its administration, which can be challenging in those with an ultimate diagnosis of ASD. A cognitive factor, however, has been reported in previous studies looking at autism screening and diagnostic measures which highlights the complexity of autism clinical presentations and developmental delays. This may be especially important in those children from different cultures (Esler et al. 2017; Stone et al. 2004). Previous studies have also shown that children of immigrants from countries with a low human resource index have a higher incidence of intellectual disability and ASD compared to other groups (Esler et al. 2017). Thus, it is essential that autism evaluations include medical and developmental history, clinical presentation, screening results, and necessary additional diagnostic testing to provide the best-estimate clinical diagnosis.

### Determination of Best Cutoff Score

In this current study, a cutoff score of 15, as initially suggested by Choueiri and Wagner (2015), or a cutoff score of 14 as suggested by Lemay et al. (2020), have similar PPV of 98%. A score of 16 or higher has a 100% PPV for a diagnosis of ASD.

**Fig. 2:**
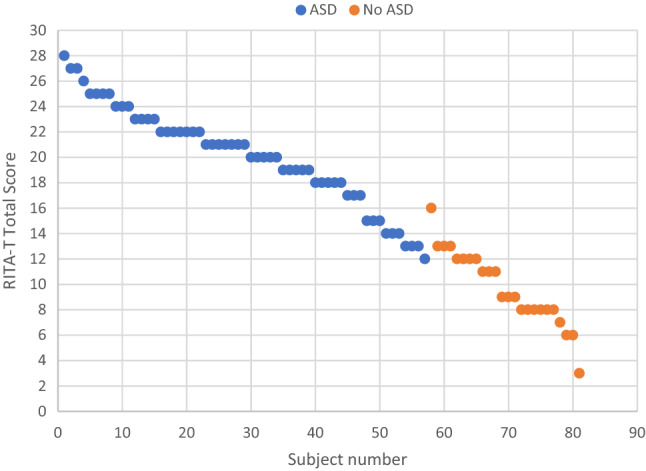
Scatterplot of RITA-T total score by diagnosis (n = 81)

In their study evaluating 239 toddlers, Lemay et al. (2020), further classified those with a cutoff score below 12 as low risk, those with a score between 12 and 16 as medium risk and those with a score above 16 as high risk. Lemay et al. then completed all testing including the ADOS only on those in the medium risk group i.e. those with a score between 12 and 16. In this current study, a score of 12 had a PPV of 93% for a diagnosis of ASD and a score of 16 had a PPV of 100%. In addition, a difference cutoff score of 2 points (i.e., between 14 and 16) could indicate a big difference in the rate of false positives. However, since there was only one child diagnosed with non-ASD/DD and a RITA-T score of 16, the false positive rate would be 1/81 (1.2%), which is quite low. It would be the same at the other cut points of 14 and 15. On the other hand, a cutoff score of 14 yielded 4 false negatives (4 toddlers with scores of 12 and 13 and diagnosed with ASD, Fig. 2).

Since our over-arching goal is to generalize this model to community settings, and to minimize false negatives and false positives, we further adopted the classification from Lemay et al. (2020) to consider that those with a RITA-T score < 12 are low risk. Children with a score between 12 and 16 are classified at medium risk, or in the “grey area”, and needing further careful evaluation. Those children with a score of 16 or above are at high risk for ASD. By applying this classification to our study population, we have no false negatives and a rate of 1.2% of false positives, the one toddler with a score of 16 and ultimate diagnosis of non-ASD/DD. This is the current cutoff score employed for the RITA-T as we continue to use it and as it is being generalized to other settings. Furthermore, the RITA-T demonstrated similar cutoff scores for those 18–24 months and 24–36 months. This is in contrast with the STAT (Screening for Autism in Toddlers) where cut off scores are different for those younger than 24 months (Stone et al. 2008). Because it involves little need for child language skills, it is also easily applicable for use with young children in culturally diverse communities. It is also more useful with all children with little or no language. As always, expert clinical judgement remains essential when evaluating toddlers with this measure, or with any other screening measure.

The initial paper by Choueiri and Wagner (2015) included a third group of toddlers with no developmental concerns. The initial paper was exploratory to determine valid cutoff scores and required that third group for comparison. It was clear that the “no developmental concerns” group performed similarly to the non-ASD/DD group and differently than the ASD group. Thus, the focus after this initial paper was the integration and the performance of the RITA-T in different clinical and community settings. Specifically, we sought to empirically differentiate between those children with ASD and referred children who were non-ASD/DD.

Partnering with EI providers is important and valuable in a variety of ways. Frontline EI professionals have already built a relationship of trust with the families they serve, and they use this rapport to broach concerns more sensitively with families. It is important to note that the PPV of the MCHAT R/F for ASD in this project (87.7%) is much higher than what is reported in the literature and by other studies. This finding likely was due to the fact that our sample was composed of children more carefully screened initially: EI providers involved with the study went over the questions in detail with parents and demonstrated behavioral features to ensure better comprehension. This reinforces the need for providers to go over the MCHAT R/F questions with the family completing them in detail, and to administer the follow-up interview when required. This recommended approach yielded a much better PPV and overall improved early detection. It also set the stage for parental comprehension and eventual acceptance of the diagnosis when given.

Early intervention professionals can gradually share concerns for ASD through screening, facilitating referral of children and parents to appropriate diagnosticians, and they often accompany families to the evaluation. All these factors serve to support the challenging process of delivering a difficult diagnosis to parents. In addition, young children from underserved communities may not have a consistent primary care provider or healthcare site, eliminating a continuity of care which allows for frequent monitoring of child development over time. EI providers will routinely observe and monitor a child’s progress in areas of concern.

In summary, this demonstration study shows that the RITA-T is a low-cost, reliable Level 2 screening test, and its integration in a two-level screening model was found to be feasible, valid and easily completed in a community setting. While we do realize this is a small number of toddlers, and in a particular geographic area and with a particular EI program, results are encouraging for its generalization to other community settings. Another possible limitation of the study was that the PI and research team were involved in the diagnostic process. However, great care was taken to keep the investigators blinded to the screening results. It will be important to seek replication of our findings by other researchers in a range of real-life settings and different populations of children and families.
